# Risk factors for some tropical diseases in an African country

**DOI:** 10.1186/s12889-021-12286-3

**Published:** 2021-12-11

**Authors:** F.-M. E. Uzoka, C. Akwaowo, C. Nwafor-Okoli, V. Ekpin, C. Nwokoro, M. El Hussein, J. Osuji, F. Aladi, B. Akinnuwesi, T. F. Akpelishi

**Affiliations:** 1 Dept. of Math and Computing, Mount Royal University, 4825 Mt Royal Gate SW, Calgary, AB T3E 6K6 Canada; 2 Dept. of Public Health, University of Uyo Teaching Hospital, Uyo, Nigeria; 3 Canadian Institute for Innovation and Development, Calgary, Canada; 4 Morat Medical Centre, Benin City, Nigeria; 5 Dept of Computer Science, University of Uyo, Uyo, Nigeria; 6 School of Nursing, Mount Royal University, Calgary, Canada; 7 School of Nursing, Mount Royal University, Calgary, Canada; 8 Health Watch Medical Clinic, Calgary, Canada; 9 Dept of Computer Science, University of Eswatini, Kwaluseni, Eswatini; 10 Health Centre, Bells University of Technology, Otta, Nigeria

**Keywords:** Risk factor, Infectious diseases, Tropical diseases, Communicable diseases and sub-Saharan Africa

## Abstract

**Background:**

Often, non-clinical risk factors could affect the predisposition of an individual to diseases. Understanding these factors and their impacts helps in disease prevention and control. This study identified risk factors for malaria, yellow fever, typhoid, chickenpox, measles, hepatitis B, and urinary tract infection in a population in an African country.

**Methods:**

Our study was an observational, correlational, and quantitative one that explored relationships among risk variables and disease prevalence - without modifying or controlling the variables. Data for this study was obtained through random sampling of a population of patients and physicians in the eastern/southern, western, and northern parts of Nigeria in 2015–2016. A total of 2199 patient consultation forms were returned by 102 (out of 125) physicians, and considered useful for analysis. Demographic data of patients, physicians, and diagnosis outcomes were analysed descriptively through frequency distributions, aggregate analysis, and graphs. The influence of risk factors on the disease manifestations (diagnosis outcomes) was determined using regression analysis.

**Results:**

Our results show that living in a tropical climate is by far a major risk factor associated with tropical diseases (malaria: t = 19.9, typhoid: t = − 3.2, chickenpox: t = − 6.5 and typhoid: t = 12.7). The risk for contracting infections is relative to specific diseases; for example, contact with chickenpox infected person poses a high risk of contracting the virus (t = 41.8), while poor personal hygiene predisposes people to high risk of urinary tract infection (t = 23.6). On the other hand, urbanization and homelessness pose very low risks of disposing the individual to the diseases under consideration, while low fluid intake, lack of voiding, and wearing non-cotton underwear predispose individuals to few diseases.

**Conclusion:**

The risk factors identified in our study exert differential and discriminating influences in the causation, predisposition, and transmission of these disease studied. It is recommended that significant effort be devoted by governments in the tropics to the mitigation of these modifiable risk factors. The most important strategy to mitigate the occurrence of these risk factors will be improving the living conditions of people and the provision of social protection measures to reduce the occurrence and burden of these diseases.

## Background

Tropical diseases are a diverse group of infectious diseases that are endemic in tropical and subtropical regions. They are caused by pathogens like viruses, bacteria, and parasites. Unfortunately they tend to be ‘neglected’ as they typically affect people in low socioeconomic classes, with poor sanitary and housing conditions as well as people in areas that have difficult access to health services [1]. Tropical diseases represent a significant public health problem and have high mortality rates especially if left untreated [1, 2]; as such, tropical diseases have drawn the attention of international health organizations.

Due to globalization of trade and increased travel to and from endemic regions tropical diseases are no longer localized in tropical regions [3]. The difficulty associated with early diagnosis is of particular concern because tropical diseases can present with overlapping and confusable symptoms. Understanding the symptoms, risk factors, diagnosis, and treatments of these diseases is of global importance as they can give further insights into their differential diagnoses. Tropical diseases present a diagnostic challenge to physicians, especially in non-endemic regions. An understanding of the various risk factors for tropical diseases can give improved insights into their differential diagnoses. Besides, research has shown that risk factors for a particular disease could change over time. It has been established that a substantial epidemiological shift occurred in the contribution of different risk factors to disease burden within 1990–2010 [4].

About two decades ago, communicable, maternal, perinatal, and nutritional disorders explained 43.9% of all causes of death and disability, while non-communicable causes explained 40.9% [5]. This picture has changed drastically; in 2017, the leading causes of death and disability worldwide had moved from communicable towards non-communicable diseases [6]. Evidence shows that between 1990 and 2017, early death from enteric infections, respiratory infections and tuberculosis, and maternal and neonatal disorders dropped, with the greatest declines in the least developed countries [6]. However, these broad global disease patterns mask enormous regional and geographical variation in health risks [7]. In sub-Saharan Africa, risks such as childhood underweight, household air pollution, and micronutrient deficiency continue to cause a disproportionate health burden [6].

Malaria is one of the most common tropical diseases with about 3.2 billion of the world’s population at risk of the disease [8]. An efficient system for malaria transmission requires strong interaction between the hosts, the ecosystem and infected vectors as it is spread through the bites of infected mosquitoes [9, 10]. Another tropical disease of significant public health importance is typhoid fever, also called enteric fever. It is a systemic bacterial illness transmitted from person to person via the fecal-oral route [10]. Several factors have been linked to risk of having typhoid fever including domestic use of contaminated water, poor waste management, and living in urban slums [11–13]. Similar to malaria, yellow fever which is endemic in tropical and subtropical areas of Africa and South America is also spread through the bite of infected mosquitoes. Hence its risk factors are related to factors that favour vector proliferation and those that reduce host immunity [14–17].

Several other diseases including measles, varicella zoster, urinary tract infections and hepatitis B can present with confusable symptoms. Although these are not limited to the tropics, they sometimes present atypically or with increased severity in tropical regions and are therefore a cause for concern. For example, Varicella zoster, which typically causes mild disease in children [13, 14], occurs at a later age in the tropics where it causes a more severe and sometimes fatal disease [15]. Also, several practices predispose those in some African countries to a higher risk of Hepatitis B which is a blood borne disease. Examples of these practices include traditional scarification and tattooing, circumcision by traditional healers, and female genital mutilation. This is because instruments with poor standard sterilization measures are typically used and reused [16].

The risk factors for tropical diseases are different from those of non-communicable diseases (NCDs) in that the risk of non-communicable diseases are mainly related to individual lifestyle and often reflect the choices of persons such as physical inactivity, high calorie diets, alcohol use and cigarette smoking [6]. For communicable diseases, however, an assessment of the risk factors helps to narrow down from the array of confusable signs and symptoms, bringing to focus the index disease for diagnosis. Assessing risk factors for diseases also gives the opportunity for risk factor modification in order to prevent future recurrences of these diseases [17]. Understanding these risk factors at individual and population levels will lead to informed policy, decision making, and planning accurate interventions.

The purpose of this study was to identify risk factors for malaria, yellow fever, typhoid, chickenpox, measles, hepatitis B and urinary tract infection (UTI) in Nigeria, the most populous African country. We analysed risk factors associated with some tropical diseases that are prevalent in the country. We also determined the prevalence of these diseases in the population based on diagnoses.

## Methods

### Study design

Our study was an observational, correlational, and quantitative (analytical) one that sought to describe and discover relationships among risk variables (independent variables) and disease prevalence (dependent variable) - without modifying or controlling the variables. We assessed the effects of exposure of the individual to the following non-clinical risk factors on disease prevalence (inferred from patient diagnosis results): low socioeconomic status (Lwsocec), immigration (Immig), tropical climate (Tropclim), urbanization - newcomers (Urbanzatn), street vendor (Strtvend), sewage contamination (Swgcont), poor personal hygiene (Popershy), overcrowding (Ovrcrwd), homelessness (Hmlsness), travel to endemic region (Trendreg), skin puncture procedure (Sknpunproc), direct contact with infected persons (Continfect), low fluid intake (Lowfliud), non-cotton underwear (Noncotund), and lack of voiding (Lackvoid).

Our sampling method was random from a population of patients in the eastern/southern, western, and northern parts of Nigeria. The patients volunteered for the study at the reception desks of their physicians, were randomly selected from hospitals and clinics in selected cities in Nigeria. The cities were selected based on convenience of access.

### Data collection

Data for this study was obtained through patient diagnosis forms, distributed to physicians in Nigeria between 2015 and 2016. Twenty patient consultation forms were distributed to each of 125 physicians, who were randomly selected from northern, western and eastern/southern Nigeria. The patient data collection form had five sections: Section A contained demographic information about the patient and the attending physician; Section B obtained information about clinical presentations (symptoms) and risk factors based on patient consultation; Section C gave the physician the opportunity to indicate suspected diseases (hypotheses); Section D contained further investigation information, while Section E showed the final diagnosis. The final diagnoses were arrived at through further diagnostic tests conducted on the patients to determine the actual disease(s) and their intensity levels. The survey instrument was pilot tested on a subset of participants. These pilot data were analysed using the principal component analysis to reduce data dimensionality and eliminate redundant variables.

Ethical clearance was sought and obtained from Mount Royal University Human Research Ethics Board. Patient written consent was sought before the utilization of the data collection form during patient consultation. All patients from the population were included in the study, in so far as they could provide consent or assisted consent plus assent (for children and other vulnerable patients). The physicians that were included in the study were general practitioners and family physicians. Specialist physicians and physicians in training were excluded from the study. There was no age or sex restriction regarding patients who participated in the study; however, to ensure that minors were protected from undue influence, only minors (ages 0–17) whose parents consented to participation in the study were included. In addition, a minor’s assent was required to participate in the study. Data collection was entirely anonymous, and completed forms were returned without any respondent’s or patient’s identification information.

### Data analysis

The Statistical Package for Social Sciences (SPSS) version 20.0 was utilized in the analysis of data. Demographic data of patients, physicians, and diagnosis outcomes were analysed descriptively through frequency distributions, aggregate analysis, and graphs. We conducted multiple linear regression analysis to determine the impact of risk (non-clinical) factors on the prevalence of tropical diseases under consideration.

A weighting of the disease prevalence by the physicians was carried out using the aggregation formula adapted from Uzoka and Ndzinge (2009) [18]:

1 $$Weighted\ Prevalence=\frac{\sum_{i=1}^5{w}_i{p}_i}{5}\kern1.75em$$

where w_i_ is the weight assigned to i^th^ level of intensity of attack of a given disease. The intensity ranges from very low (w_i_ = 1) to very high (w_i_ = 5); Pi is the percentage of patients diagnosed with the given disease, with intensity of attack i. Note that some patients were diagnosed with multiple diseases at different levels of intensity of attack. The prevalence percent is computed relative to all the weighted prevalence values.

SPSS also computes the collinearity statistics (the tolerance and variance inflation factor [VIF]) to determine if two variables are near perfect linear combinations of one another. As the degree of collinearity increases, the linear regression model estimates of the coefficients become unstable. The “tolerance” is an indication of the percent of variance in the predictor that cannot be accounted for by the other predictors, hence very small values indicate that a predictor is redundant. Ideally the tolerance should be greater than 0.5, while the VIF should be 5 or less.

## Results

A total of 2159 diagnosis forms out of 2500 (86.36%) were returned and considered useful for analysis. Table 1 shows the demographic information on the patients, the physicians who conducted the diagnosis and the weather season when the patient consultation /diagnosis was conducted. The sample consisted of 52.6% females and 47.4% males, who were mostly below 40 years (76.5%), while most of the diagnosis (63.4%) were carried out by experienced physicians (over 5 years). The levels of experience of the physicians point to their ability to clearly understand the dynamics of tropical diseases. The final diagnosis outcomes were arrived at through further tests that were conducted on the patients to determine the actual disease(s) and their intensity levels. Sixty percent of the diagnoses were conducted in the dry season, while the rest were either in the rainy or harmattan seasons. There was a shortfall of 11 in the patient sex, resulting from the sex box of some forms not checked by some physicians. Also, note that the diagnosing physician’s experience was only obtained from the patient’s diagnosis form as the data was not aggregated by diagnosing physician.

**Table 1:** Demographic

	Frequency	Percent
Sex		
Male	1038	47.4
Female	1150	52.6
Patient age (years)		
≤10	438	19.9
10–20	401	18.2
21–30	555	25.2
31–40	290	13.2
41–50	288	13.1
51–60	111	5.0
> 60	116	5.3
Diagnosing physician’s experience		
< 5	37	36.6
5–10	26	25.7
11–15	13	12.9
16–20	3	3.1
> 20	22	21.7
Season		
Rainy	381	17.3
Harmattan	655	29.8
Dry	1163	52.9

Table 2 shows the weighted and unweighted disease prevalence as diagnosed by the physicians. The highest unweighted prevalence being malaria (61.3%), UTI, (19.1%), typhoid (18.3%). However, the prevalence of the diseases differed significantly when weighted. Note that some patients were diagnosed with multiple diseases at different levels of intensity of attack. The prevalence percent was computed relative to all the weighted prevalence values. Table 2 shows the overall prevalence and intensities.

**Table 2:** Disease prevalence based on diagnosis

Disease	Intensity of attack						Weighted prevalence	Prevalence percent
	Percent diagnosed	Very low	Low	Moderate	High	Very High		
Malaria	61.30	3.40	8.00	14.10	25.10	10.70	43.12	47.91%
Typhoid	18.30	0.80	3.00	9.00	3.70	1.70	11.42	12.69%
Chicken Pox	7.20	0.10	0.20	4.60	2.10	0.30	4.84	5.38%
Measles	1.30	1.20	0.10	0.00	0.00	0.00	0.28	0.31%
Hepatitis B	3.30	1.00	0.40	1.70	0.10	0.10	1.56	1.73%
Yellow Fever	0.70	0.10	0.20	0.10	0.40	0.00	0.48	0.53%
UTI	19.10	3.10	0.50	0.90	7.80	6.70	14.30	15.89%
Other	18.40	0.00	2.70	3.20	7.50	5.00	14.00	15.56%

The results indicate that malaria was the most commonly diagnosed disease, followed by UTI, and typhoid. Malaria also with had the highest intensity of 14.1 moderate, 25.1 high and 10.7 very high. A total of 61.32% of the patients were diagnosed with malaria; 49.9% of which had moderate to very high intensity of attack, while 11.4% had low intensity. The second and third in level of prevalence were UTI and typhoid fever, with intensity weights of 14.32 and 11.50 respectively.

### Regression analysis

Table 3 shows the regression statistics. The risk factors (predictor) were regressed against the intensity of attack of each disease (outcome). The risk factors are abbreviated (first column of Table 3 because of table space and formatting constraints), while a key is provided at the bottom of the table to show the full meanings of the abbreviations. The collinearity statistics (Tolerance [Tol] and variance inflation factor [VIF]) show good levels of tolerance [> 0.2] and variance inflation factor (VIF) [< 5] for most of the predictors, relative to each disease (except for *low fluid* and *lack of void*, both of which exhibit Tol of 0.18 and 0.2 respectively). The predictive power of the model (R^2^_,_ adjusted R^2^) was generally low.

**Table 3:** Regression statistics

	Malaria		Typhoid		Chk Pox		Measles		Hepatitis B		Yellow Fever		UTI		Others		*Tol*	*VIF*
R^2^	0.491		0.366		0.647		0.023		0.033		0.037		0.465		0.286			
Adjusted R^2^	0.487		0.362		0.644		0.016		0.026		0.030		0.461		0.281			
	*t*	*p*	*t*	*p*	*t*	*p*	*t*	*p*	*t*	*p*	*T*	*p*	*t*	*p*	*t*	*p*		
Lwsocec	7.9	0.00	0.2	0.00	−6.9	0.00	1.5	0.13	0.9	0.36	−2.8	0.01	−1.3	0.19	6.8	0.00	0.56	1.78
Immig	7.8	0.00	7.2	0.81	15.1	0.00	−1.2	0.23	−3.0	0.00	0.3	0.79	1.8	0.07	−10.6	0.00	0.64	1.57
Tropclim	19.9	0.00	−3.2	0.00	−6.5	0.00	−4.3	0.00	−3.6	0.00	−3.1	0.00	12.7	0.00	−4.5	0.00	0.82	1.22
Urbanzatn	1.1	0.28	3.4	0.00	−6.7	0.00	−1.7	0.09	−2.9	0.00	1.0	0.31	1.5	0.14	2.9	0.00	0.53	1.88
Strtvend	−10.0	0.00	10.7	0.00	6.8	0.00	−0.5	0.65	−3.7	0.00	7.9	0.00	−2.7	0.01	2.6	0.01	0.69	1.44
Swgcont	4.5	0.00	10.7	0.00	−10.1	0.00	0.0	0.98	2.7	0.01	−2.0	0.05	−10.9	0.00	3.6	0.00	0.46	2.17
Popershy	−0.6	0.55	−3.5	0.00	−2.4	0.01	−3.5	0.00	−0.3	0.76	0.0	0.99	23.6	0.00	0.4	0.67	0.48	2.09
Ovrcrwd	−5.2	0.00	3.1	0.00	5.6	0.00	2.3	0.02	2.1	0.03	1.1	0.26	−1.8	0.07	11.2	0.00	0.65	1.54
Hmlsness	0.3	0.75	7.6	0.00	−0.1	0.88	−0.3	0.79	−2.2	0.03	−0.2	0.83	−5.0	0.00	−3.2	0.00	0.57	1.74
Trendreg	11.0	0.00	−10.5	0.00	−7.3	0.00	−2.3	0.02	1.4	0.17	−0.9	0.38	−5.2	0.00	6.3	0.00	0.75	1.33
Sknpunproc	−3.2	0.00	0.3	0.00	3.6	0.00	− 0.5	0.65	4.0	0.00	−1.4	0.15	−3.3	0.00	− 10.0	0.00	0.83	1.21
Continfect	−19.9	0.00	−12.8	0.76	41.8	0.00	−0.8	0.41	1.3	0.21	−1.9	0.06	−4.3	0.00	10.2	0.00	0.66	1.52
Lowfliud	−12.4	0.00	6.2	0.00	2.8	0.01	0.1	0.93	1.2	0.24	−0.4	0.68	−2.6	0.01	0.8	0.45	0.18	5.57
Noncotund	−10.9	0.00	−3.6	0.00	−1.1	0.25	−0.3	0.79	−1.0	0.30	−1.8	0.07	8.6	0.00	−3.1	0.00	0.66	1.52
Lackvoid	12.1	0.00	−8.1	0.00	−0.9	0.39	−0.2	0.86	−0.9	0.34	−0.1	0.89	10.9	0.00	−2.8	0.00	0.20	5.02

Key: low socioeconomic status (Lwsocec), immigration (Immig), tropical climate (Tropclim), urbanization - newcomers (Urbanzatn), street vendor (Strtvend), sewage contamination (Swgcont), poor personal hygiene (Popershy), overcrowding (Ovrcrwd), homelessness (Hmlsness), travel to endemic region (Trendreg), skin puncture procedure (Sknpunproc), direct contact with infected persons (Continfect), low fluid intake (Lowfliud), non-cotton underwear (Noncotund), and lack of voiding (Lackvoid)

The t-values show that the risk factors do significantly predispose individuals to infectious diseases, especially malaria, typhoid, chicken pox, and UTI. Table 4 provides a visual representation of the risk levels associated with each risk factor. This is based on the t-values in Table 3; the key to the decision on the level of risk is shown beneath Table 4. Living in a tropical climate is evidently a major risk factor associated with tropical diseases. Being a street vendor predisposes people to infection; so is overcrowding and traveling to endemic regions. It is also instructive to note that some factors pose higher risks than others; for example, contact with an infected person poses a high risk of chicken pox infection (t = 41.8 [***]), while poor personal hygiene predisposes people to high risk of urinary tract infection (t = 23.6 [***]). On the other hand, urbanization and homelessness, pose very low risks [*] across all the diseases, while low fluid intake, lack of void, and wearing non-cotton underwear only predispose individuals to few diseases (3 in each case).

**Table 4:** Disease-risk factor relationships

	Malaria	Typhoid	Chicken Pox	Measles	Hepatitis B	Yellow Fever	UTI	Others
Lwsocec	*	**	*			*		*
Immig	*		**		*			**
Tropclim	**	*	*	*	*	*	*	*
Urbanzatn		*	*		*			*
Strtvend	**	*	*		*	*	*	*
Swgcont	*	**	**		*		**	*
Popershy		**	*	*			***	
Ovrcrwd	*	*	*	*	*			**
Hmlsness		*			*		*	*
Trendreg	**	*	*	*			*	*
Sknpunproc	*	**	*		*		*	*
Continfect	**		***				*	**
Lowfliud	**	**	*				*	
Noncotund	**	*					*	*
Lackvoid	**	*					**	*

**Key**

**Table:** 

Risk Level	Condition	Symbol
Low	2 ≤ t < 10	*
Medium	10 ≤ t ≤ 20	**
High	t > 20	***

## Discussion

This study aimed to identify risk factors for selected diseases in Nigeria, the most populous African country. We analysed risk factors associated with some tropical diseases and determined the prevalence of these diseases in the population based on diagnoses. We found the diseases with the highest unweighted prevalence to be malaria (61.3%), UTI (19.1%), typhoid (18.3%); however, the prevalence of the diseases differed significantly when weighted. Regression analysis confirmed that these risk factors do significantly predispose individuals to infectious diseases, especially malaria, typhoid, chickenpox, and UTI. Furthermore, some factors posed higher risks than others; for example, contact with an infected person poses a high risk of chickenpox infection, and poor personal hygiene predisposes people to high risk of urinary tract infection. On the other hand, urbanization and homelessness pose very low risks across all the diseases, while low fluid intake, lack of voiding, and wearing non-cotton underwear predispose individuals to few diseases.

The study focused on epidemic prone and priority diseases in Nigeria [19, 20], including malaria, typhoid, chicken pox, UTI, measles, yellow fever and hepatitis B. These tropical diseases are diseases of public health importance in Nigeria. Surveillance of these diseases of public health importance is captured by using the Integrated Disease Surveillance and Response (IDSR) [21]. IDSR is a framework implemented to improve detection and response to the primary causes of morbidity and mortality in African countries. However, there have been challenges to the implementation of this policy, one of which is the limited capacities of personnel to identify and report IDSR priority diseases [22, 23]. This underscores the importance of our study to enhance the capacity of health workers to identify and report these diseases for adequate surveillance.

Our results indicate that malaria, UTI, and typhoid are prevalent in Nigeria and agree with previous works done on disease prevalence in the country [1, 24, 25]. These diseases remain diseases of major public health importance in Nigeria. Although UTI is not classified as a tropical disease, the burden of the disease in Nigeria and the fact that some of the causative organisms could be tropical led to its inclusion in the index study. It has been shown previously that malaria and typhoid are at the top of the chart of the most overwhelming health problems facing the tropical and subtropical African countries, with malaria alone claiming 213 million cases and over 380,000 deaths per year [26]. It is reported that a 1 °C increase in maximum temperature in a given month was related to a 15 and 19% increase in malaria incidence in the same and subsequent month, respectively [27].

Similar to findings in our study, a study in central Vietnam linked low socioeconomic class to be an important risk factor for malaria. It was opined that this was possible because of poorer housing conditions increasing their exposure to infective bites within their villages and the fact that those of lower socioeconomic class were more likely to make regular visits to and even sleep in forests [28]. Poverty can therefore be said to be an underlying risk factor, linking and potentiating the different risk factors for malaria [29]. Higher socioeconomic status is associated with better housing structures, the situation of houses in better environments, the ability to afford prevention methods, and better refuse collection, among others, all of which reduce the risk of malaria infection [30].

In our study, the majority of the cases of malaria diagnosed was high intensity, and for typhoid, moderate intensity. This highlights the public health significance of malaria as an endemic disease in the study areas. The majority of the physicians who conducted the study were urban-based; this could explain the high intensity and transmission. It has been observed that contrary to the expectation that urbanization should reduce malaria transmission, the disease still persists in African cities, in some cases at higher levels than in nearby rural areas. The explanation was suggested to be an adaptation of malaria vector species to the urban environment [30].

The study also highlights typhoid as a major public health disease. Several factors have been linked to risk of having typhoid fever. Studies have linked domestic use of contaminated water to the incidence of enteric fever [10, 11]. This is important in Nigeria as only 64.1% of the population have household members with improved access to drinking water and the percentage of household members using improved sanitation facilities which are not shared is 35.1% [31]. These factors predispose people to use fecally-contaminated water for food preparation and drinking, thus placing them directly at risk for typhoid fever. In addition, consumption of raw vegetables, meat, seafood, and milk, which are often contaminated with excreta of an infected person, has been shown to increase the risk of typhoid fever [11, 32]. This can be linked to the consumption of street foods [33]. Akuu et al. reported that those who ate out in food stalls were 6.9 times more likely to have typhoid fever [34]. This finding was attributed to poor hygiene maintained by commercial food vendors as they have little to no regulation in most tropical regions [34, 35].

This study revealed some critical risk factors linked to the diseases studied. *Living in a tropical climate* is evidently a major risk factor associated with tropical diseases in Nigeria. This is a risk factor that is clearly non-modifiable except by migration; therefore, this can be termed as an overarching risk factor that sets the stage for interplay of the other risk factors. This finding can be understood from the standpoint of the lifecycle of the causative agents of the diseases where warm climate plays a major role in the reproduction and propagation of the pathogens. It has been shown that temperature and moisture variables are the most important drivers for some tropical diseases like malaria [27, 36]. Rapid urbanization seen in our cities with attendant destruction of the natural environment is associated with this risk factor [37, 38]. Thus, poor urban structures with the creation of urban slums expose people to more pathogens, creating a vicious cycle of poverty and disease.

Another important risk factor is *travel from a non-endemic to an endemic region*, which increases the risk of contracting malaria as individuals with no natural immunity are inevitably exposed to the disease in these areas [39]. The travellers are also at risk of eating unsafe foods and drinking contaminated water, underscoring the importance of abiding by food safety regulations.

We also found that the factors that measured the level of exposure to infectious agents were important predictors for diseases. It was found that *being a street vendor* predisposes people to infection. This finding is expected because this risk factors enhances contact with disease pathogens and possible disease establishment. As observed in this study, contact with an infected person increased the risk of chickenpox by about 42 times. In the case of UTI, poor personal hygiene leads to exposure and prolonged contact with infectious agents. Poor hygiene therefore increased the risk of UTI up to about 24 times as seen in this study. A previous study also reported the link between poor genital hygiene and development of UTIs [40].

It is also important to note that the levels of risk factors in our study were found to vary, with some risk factors predisposing individuals to more diseases than others. Among the factors with low-risk levels were *urbanization*, *low fluid intake*, *lack of voiding*, and *wearing non-cotton underwear* predisposing individuals to few diseases. However, we were surprised to observe that *homelessness* also predisposed individuals to only few diseases. Previous studies have shown that homelessness predisposes individuals to a host of diseases [41–43]. We can attempt to explain this variation by looking at the geographical location where these studies were conducted.

Many of these diseases are preventable and/or treatable through specific interventions [44]. Although various pathogens cause the vast array of tropical diseases, the risk factors that are commonly seen include low socioeconomic status, poverty and overcrowding. With globalization and urbanization, these risk factors are compounded as the urban slums are characterized by homeless people, street vending, poor personal hygiene, close contact with infected persons as well as poor sanitary conditions. With progressive underinvestment in infrastructure and amenities, there are disproportionate levels of poverty and poor living conditions [45, 46]. Compounding these is poor access to affordable healthcare services in Nigeria.

Access to health services especially for the low socioeconomic class is poor, with only about 5% of Nigerians covered by the national health insurance scheme under the NHIS [47]. This leaves a majority of the urban poor without access to preventive health care and continuous exposure to these risk factors compound the existing poor health status leading to worsening morbidity and mortality. In addition, health resource allocation is in favour of secondary and tertiary care, while primary health care which attends to a majority of the people are poorly funded. This leads to people seeking primary care at the secondary and tertiary centres. Therefore, it is critical to reorient the health sector towards promoting health, especially at the primary care level, by strengthening the PHC delivery system and reducing health inequities as espoused by the WHO. This would improve access to preventive health services, thereby curbing the spread of some of these tropical diseases.

### Study implications

The majority of the risk factors studied here could be termed as proximal determinants of health. Interestingly, we have noted that they are largely modifiable. Although not traditionally classified as modifiable in communicable disease epidemiology, we make a case for treating these risk factors as modifiable. This is pertinent against the background of the evidence that drivers of poor health can be reduced by addressing the social causes of poor health. Therefore, improving the living conditions of people including; urban infrastructure and governance, early childhood development and education, employment conditions, and social protection measures, will improve populations’ health and well-being. Addressing the social determinants related to tropical diseases is a reasonable public health intervention for disease control and at the same time, is a prerequisite for confronting inequities in health [7]. Further studies on how homelessness influence disease acquisition in tropical settings, especially in sub-Saharan Africa, are required to understand the true picture of the risk factor in tropical countries.

### Limitations

We acknowledge the limitations of this study as follows: Some key tropical diseases that are common in Sub Saharan Africa were not included in the study, including tuberculosis and upper and lower respiratory tract infections. Also, our study focused solely on Nigeria as a geographical entity for data collection. Although this limits the generalizability of study, we expect that our findings can be extrapolated to other regions of Africa and the tropics, since the risk factors focused on are similar in these areas. Additionally, we recognize that variables included in this study are limited to mostly environmental (non-medical) circumstances. A future study will include personal(medical) risk factors, such as blood pressure, high cholesterol level, genetic conditions and allergies.

### Recommendations

There is a need to reduce inequities and inequalities in society by addressing the poverty and low socioeconomic status of the urban populations. We recommend that significant effort be put into mitigating some of these factors, which are low socioeconomic class, homelessness, overcrowding as well as the negative effects of urbanization. This can be done by government strategies targeting improving living conditions, provision of potable water and sanitation facilities, and provision of housing. Furthermore, poverty, which was seen to be an underlying factor potentiating these risk factors, should be addressed by the creation of job opportunities for people both in the public and private sector.

At the individual level, poor hygiene, poor voiding, wearing of non-cotton underwear, and low fluid intake at the individual level can be achieved through engagement at the community level with various public health interventions targeted at behavioral changes. Public Health Specialists and other health officials have the tasks of engaging the communities to provide health education, raising awareness of these risk factors, and its effect on health to effect the necessary behavioral change.

Finally, the federal ministry of health should ensure vaccination and chemoprophylaxis of non-immune individuals migrating to or visiting endemic regions to prevent and reduce the severity of these tropical diseases. The findings in the present study underscore the complexity and risk factors associated with the transmission of tropical diseases and further explains why people exposed to the risk factors mentioned have higher chances of contracting some of the tropical diseases.

### Policy recommendations

Policies that address poverty and poor housing conditions in the country should be instituted. These could be done by creating affordable housing and by instituting employment programmes for the poor and most vulnerable. Furthermore, policies that mitigate indiscriminate drilling of boreholes should be enacted in order to prevent use of contaminated water.

Based on the study findings, our ultimate goal is to attempt to develop the framework for a soft computing system, which will take into consideration tropical disease risk factors and tropical disease symptoms in order to facilitate easier and more efficient differential diagnosis of these diseases.

## Conclusion

The processes involved in diagnosing and differentiating tropical disease conditions by medical practitioners are complex and may sometimes be confusing because of overlap and vagueness in symptoms presented by the different conditions. Furthermore, most of these conditions share same predisposing or risk factors. This builds the case for the exploration and understanding of the impact of different risk factors on the populations’ predisposition to these conditions.

Of the studied infectious diseases, malaria, UTI and typhoid fever were the most significant in terms of morbidity. The risk factors discussed and explored in this study also significantly influence the spread of these conditions, hence the need for evidence-based decisions to control these diseases.

The findings of this study have made a case for treating the risk factors for communicable diseases as being modifiable and linked directly with social determinants of health. Action is required at governmental, community, and individual levels to improve and mitigate these risk factors. Interventions by the order government to improve the social determinants of health associated with living conditions are key to reducing these communicable diseases’ risk factors. To this end, concerted efforts should be made by governments towards the implementation of policies to reduce inequities and inequalities associated with the social determinants of health in the country.

## Data Availability

The datasets used and/or analysed during the current study are available from the corresponding author on reasonable request.

## Abbreviations

NCD: Non-communicable disease
UTI: Urinary tract infection
SPSS: Statistical Package for Social Sciences
VIF: Variance inflation factor
IDSR: Integrated Disease Surveillance and Response
Lwsocec: Low socioeconomic status
Immig: Immigration
Tropclim: Tropical climate
Urbanzatn: Urbanization – newcomers
Strtvend: Street vendor
Swgcont: Sewage contamination
Popershy: Poor personal hygiene
Ovrcrwd: Overcrowding
Hmlsness: Homelessness
Trendreg: Travel to endemic region
Sknpunproc: Skin puncture procedure
Continfect: Direct contact with infected persons
Lowfliud: Low fluid intake
Noncotund: Non-cotton underwear
Lackvoid: Lack of voiding
